# Treatment approaches in relapsed or refractory peripheral T-cell lymphomas

**DOI:** 10.12688/f1000research.22257.1

**Published:** 2020-09-04

**Authors:** Cheryl Foster, John Kuruvilla

**Affiliations:** 1 Division of Medical Oncology and Hematology, Princess Margaret Cancer Centre, 6-424 700 University Avenue, Toronto, ON, M5G 1Z5, Canada; 2 Department of Medicine, University of Toronto, 1 King’s College Cir, Toronto, ON, M5S 1A8, Canada

**Keywords:** T-cell lymphoma, relapsed, refractory, stem cell transplant, targeted agents

## Abstract

Peripheral T-cell lymphomas (PTCLs) are a heterogeneous group of rare and aggressive non-Hodgkin’s lymphomas. Clinical staging, prognostic scoring, and initial treatment strategies have historically been based on paradigms developed in B-cell lymphomas. Despite primary treatment protocols that are typically anthracycline-based and frequently involve consolidative autologous stem cell transplantation in first remission, many patients develop disease progression. There remains a high unmet medical need for improved treatment strategies in the relapsed or refractory setting. Salvage chemotherapy and stem cell transplantation in those who are suitable has traditionally been the accepted approach, but this remains a minority of the total patient population. As increasing knowledge is gleaned regarding the biological heterogeneity within the various PTCL subtypes, newer targeted agents have been developed, studied, and approved in this small, heterogeneous population of relapsed or refractory disease. Given its success and tolerability in this pretreated population, brentuximab vedotin, an anti-CD30 antibody drug conjugate, was brought earlier in the disease course and is a model for advances in the targeted treatment of PTCL. As others undergo further development in the relapsed setting and successes are brought earlier in the disease course, the outcome for PTCL patients is likely to improve. However, innovative clinical trial designs are crucial for the assessment of targeted agents in this highly heterogeneous population. This review explores the current treatment environment for patients with relapsed and refractory PTCL, including newer strategies such as targeted agents and immunotherapy.

## Introduction

T-cell lymphomas are a heterogenous group of uncommon non-Hodgkin’s lymphomas (NHLs) which account for approximately 10–15% of all NHLs. The prevalence varies geographically, with the highest incidence in Asia, particularly of natural killer (NK)/T-cell lymphoma and adult T-cell leukemia lymphoma (ATLL) due to variations in exposure and susceptibility to HTLV-1 and EBV. Subtypes which arose from mature T-cells were classified as peripheral T-cell lymphomas (PTCLs) and could be subdivided into clinically indolent or aggressive lymphomas. Historically, these variations in clinical behavior rather than a pathologic basis determined the therapeutic approach. Treatment protocols were chosen to treat aggressive PTCLs owing to their success in treating aggressive B-cell lymphomas; however, outcomes were not as successful. It is amongst the aggressive group of PTCLs that the most recent WHO classification has incorporated significant advances in the knowledge of the biological heterogeneity of PTCLs to better classify and describe new entities which may help to explain the clinical heterogeneity of these aggressive lymphomas. The relapsed and refractory (RR) setting remains an important population with aggressive disease in which to explore novel targeted approaches prior to moving them into the frontline setting and addressing an unmet need in NHL treatment.

## Primary therapy

Established upfront therapies in the treatment of aggressive PTCL include anthracycline-based chemotherapies, such as CHOP (cyclophosphamide, doxorubicin, vincristine, and prednisone) and stem cell transplantation (SCT), with this approach adapted mostly from success in treating aggressive B-cell NHLs. The International T-cell Lymphoma Project examined a cohort of 1,314 cases of PTCL and NKTCL and while cases of ALK-positive anaplastic large cell lymphoma (ALCL) had a 70% 5-year overall survival (OS), the remaining subtypes had worse outcomes, with 5-year OS for PTCL-NOS, angioimmunoblastic T-cell lymphoma (AITL), and all NKTCLs of 32% and a more dismal 14% for ATLL. Outcomes for ALK-negative ALCL patients were intermediate, with a 5-year OS of 49%. Thus, outcomes remain poor in the majority of subtypes and RR disease is a frequent problem for patients with PTCL ^1, 2^.

Both autologous SCT (ASCT) and allogeneic SCT (allo-SCT) have been offered to patients in both the primary and the RR setting, with ASCT being offered as consolidation in many patients undergoing frontline treatment. SCT has been established as standard of care in PTCL frequently in the absence of randomized evidence, which is challenging to achieve in rare diseases. Enteropathy associated T-cell lymphoma (EATL) is a rare subtype with dismal outcomes. A retrospective series and a small phase II study in which patients with EATL were treated with an intensive chemotherapy regimen followed by ASCT suggested improved outcomes with this approach ^3, 4^. Targeted agents have already been approved for use in PTCL, with brentuximab vedotin now established as a clear standard in the primary treatment of CD30-positive PTCL based on the randomized ECHELON-2 clinical trial ^5^. With the majority of patients in this study with systemic ALCL, it remains unclear whether brentuximab vedotin has the same effect in AITL and PTCL-NOS. A small post-hoc analysis of non-ALK-positive ALCL patients in this study favored consolidative SCT in first remission; however, sample sizes were quite small ^6^. Careful consideration must be taken during further trial designs to identify which patient or disease subgroups are most likely to benefit from brentuximab or from other targeted agents, a challenge in this rare group of diseases.

## Outcome and prognosis in RR-PTCL

There are few series that report the outcomes of RR-PTCL at a population level ^7– 9^. Data from the BC Cancer Agency reported the outcome of 153 patients with a median OS and progression-free survival (PFS) after relapse or progression of 5.5 and 3.1 months, respectively. Patients with good performance status appeared to have more favorable outcomes with standard chemotherapy ^8^. A smaller series from Modena, Italy, of 53 patients reported similarly poor outcomes with a median survival after relapse of 2.5 months ^7^. The International T-cell Project reported the results of 436 (47%) patients who were refractory and 197 patients (21%) who had relapsed out of 937 patients who had received first-line treatment ^10^. The median OS was 5.8 months, with univariable analysis showing that refractory disease (hazard ratio [HR] 1.43) and salvage therapy with SCT (HR 0.36) were associated with better survival. The only histologic subtype with a more favorable outcome was ALK-positive ALCL, which had an approximately 50% OS. All three series highlight the minority of patients undergoing SCT approaches, with only 99 patients (16%) in the population able to undergo SCT.

These three series highlight the challenges in assessing the literature in this setting. Retrospective series often require 10 to 20 years to accumulate a reasonable number of patients to report and are forced to group multiple histologies and eras into a single report. The prospective T-cell Project has the advantage of multiple centers accruing data prospectively and thus has the strength of reasonable patient numbers, even within rare subtypes of PTCL. Histology was reviewed by an expert hematopathology panel in 70% of these patients, although imaging and response assessment were performed locally with no central review, limiting some interpretation of the data.

While PTCL histology does not appear to be a major driver in outcome post failure of primary treatment (with the exception of the more favorable prognosis of ALK-positive ALCL), poor performance status and the presence of refractory disease appear to be recurrent independent predictors of inferior survival. The definition of refractory disease often varies between studies; the T-cell Project defined this as no response or progression to treatment within 1 month from the end of initial therapy or unsatisfactory partial remission (PR) according to physician judgement that would immediately require second-line treatment. There are some similarities present in the use of staging and prognostic scoring tools in PTCL, as they have largely been adapted from systems developed for use in B-cell NHLs; however, there are no uniformly accepted approaches.

The Ann Arbor staging system is commonly used despite T-cell lymphomas frequently presenting with extranodal sites of disease. PET/CT is now recommended as part of the pre-treatment staging evaluation for newly diagnosed PTCLs ^11, 12^. However, the data driving the significance of PET/CT remain restricted to smaller series. A few small retrospective studies have explored the prognostic use of interim PET/CT and end of treatment PET/CT with conflicting results ^13– 16^; therefore, interim PET/CT are not routinely done. Series incorporating PET/CT as part of response assessment in RR-PTCL remain limited. Shea and colleagues reported a cohort that did not associate a negative FDG-PET scan with favorable outcomes in patients undergoing ASCT or allo-SCT ^17^. The BC Cancer Agency and International T-cell Project series do not allow interpretation of functional imaging and relationship to outcome ^8, 10^. Interestingly the IPI and the Prognostic Index for PTCL-U (PIT) do not appear to influence outcome in RR-PTCL beyond the importance of performance status. The PIT uses the clinical characteristics of age, performance status, LDH level, and bone marrow involvement to classify patients into four prognostic risk groups ^18^; only ECOG PS appears to broadly predict outcome in RR-PTCL.

## Salvage therapy approaches

In general, there are two approaches in the setting of primary treatment failure; the first emphasizes a goal of curative therapy with a plan to proceed to SCT, while the second is typically palliative in nature and often driven by patient age, comorbidity, and/or performance status. There are no randomized trials that have specifically evaluated chemotherapy in RR-PTCL, but the CCTG LY.12 trial included PTCL in a population of patients planned for ASCT. Skamene and colleagues reported a subset analysis of the study in 59 patients with PTCL who were randomly assigned to DHAP (dexamethasone, high-dose cytarabine, and cisplatin) or GDP (gemcitabine, dexamethasone, and cisplatin) and demonstrated similar overall response rates (ORRs) (DHAP: 33%; GDP: 38%) with 1-year event-free survival (EFS) of 16% and 1-year OS of 28%. In patients going on to receive ASCT, the 2-year EFS and OS were 21% and 42%, respectively ^19^. A prospective analysis from the CISL group in Korea using GDP reported a favorable ORR of 62%. Numerically, this result looks superior to the CCTG trial, but disease and patient heterogeneity along with sample size likely contribute to some potential differences in outcome ^20^. Other regimens such as ESHAP (etoposide, methylprednisolone, cytarabine, and cisplatin) or ICE (ifosfamide, carboplatin, and etoposide) have been evaluated with similar results ^21, 22^. As such, there is no gold standard salvage therapy regimen employed for RR-PTCL.

A systematic review published in 2015 identified 14 studies using 12 different salvage approaches and included 618 RR-PTCL patients ^23^. The ORR ranged from 22–86%, incorporating a variety of targeted approaches along with single and multi-agent chemotherapies. Similarly, ORRs varied between disease subtypes from 14–72% for PTCL-NOS, 8–54% for AITL, and 24–86% for ALCL. PFS and OS ranged from approximately 2 to 15 months. This study appears to be the only systematic review in the field highlighting the lack of data guiding clinical decision-making when choosing specific treatment approaches.

Typically, conventional chemotherapy has been given prior to SCT, as “chemosensitivity” remains a core concept to demonstrate, particularly in the ASCT setting. If disease is not responding to salvage chemotherapy, then there is doubt that SCT may be beneficial. However, given the favorable single-agent activity of targeted agents in RR-PTCL and the underwhelming results of conventional therapies, targeted therapies have been used without chemotherapy, with sensitivity to the agent prior to SCT remaining an important goal. There are few datasets that have analyzed patients receiving a variety of therapies in a SCT-eligible population. A retrospective analysis from the COMPLETE dataset in RR-PTCL showed that 46% of patients received combination therapy while 54% of patients received monotherapy as a salvage approach ^24^. A total of 70% of patients were treated with “curative intent”, and these patients were more likely to receive combination therapy versus single-agent therapy (85% versus 58%). Combination regimens were typically traditional chemotherapy based (gemcitabine, ifosfamide, or platinum), while single agents were more typically newer or targeted agents (brentuximab vedotin, romidepsin, or pralatrexate). A higher proportion of patients in the single-agent group achieved complete remission (CR) (41% versus 19%) and subsequently underwent SCT (26% versus 8%), resulting in improved PFS (11 versus 7 months) and OS (39 versus 17 months).

## Stem cell transplantation

Prospective randomized trials for SCT in the relapsed setting are also lacking; however, expert consensus strongly recommends ASCT for relapsed chemosensitive disease (in most subtypes) when it wasn’t performed frontline and consideration of allo-SCT in some cases ^25^. The Parma study established the role of ASCT in the treatment of relapsed NHLs, and multiple subsequent series have been published reporting the outcomes of SCT for PTCL from institutional and registry series ^26– 33^. A systematic review published in 2016 highlights ASCT outcomes in the RR setting with a PFS of 36%, OS of 47%, relapse/progression of 51%, and non-relapse mortality (NRM) of 10% ^34^. Favorable outcomes were seen in patients with ALCL. The largest series included 241 patients with ALCL, PTCL-NOS, and AITL who underwent ASCT or allo-SCT reported to the Center for International Blood and Marrow Transplant Research (CIBMTR). ASCT patients beyond CR1 had a 3-year PFS of 42% and OS of 53%. Among allo-SCT patients who received transplant beyond CR1, 31% remained progression-free at 3 years ^31^. Rodriguez and colleagues used the GELTAMO database and identified 123 patients with relapsed or primary refractory PTCL. With a median follow-up of 61 months, the 5-year OS was 45% and PFS was 34% ^29^. A small retrospective series that included 32 patients auto-transplanted in second complete or partial response found a 5-year PFS of only 12% and OS of 40% ^32^. While difficult to undertake in a rare and heterogeneous disease, prospective studies are certainly needed in order to clarify the role of transplant in relapsed PTCL.

Schmitz recently reviewed the results of allo-SCT in RR-PTCL ^35^. In general, the results of these largely national or registry studies report similar results, with 50% of patients experiencing long-term survival with a PFS of approximately 40%. The series all have heterogeneity in patient population, donors (matched related and unrelated with haploidentical transplants being less frequently employed), intensity of preparative regimens (myeloablative or reduced intensity conditioning [RIC]), and PTCL subtypes as well as the use of *in vivo* T-cell depletion with agents such as antithymocyte globulin. RIC allograft recipients appear to have less NRM but higher rates of relapse, as has been seen in other types of lymphoma.

SCT approaches have been utilized in RR-PTCL as a standard treatment modality for many years. The safety and feasibility of these approaches have improved because of better supportive care and the adoption of RIC strategies and the use of T-cell depletion (depending on donor type). Given the preference to consider consolidation of primary treatment response with ASCT in PTCL, patients may typically flow towards allograft strategies in the relapse setting. Unfortunately, prospective comparative research has not been performed in the RR setting, though attempts have been made to perform prospective comparative trials in the consolidation of primary treatment response.

## Targeted therapies

The majority of recent advances in the management of patients with PTCL has been in the development and use of targeted agents in treating RR-PTCL. A number of drug classes including histone deacetylase (HDAC) inhibitors, monoclonal antibodies, antifolates, kinase inhibitors, and antibody drug conjugates (ADCs) have been assessed with promising results, leading to drug approval for some of these agents based on response rates in this setting ( Table 1).

**Table 1.** Summary of select trials with targeted single agents in relapsed/refractory PTCL.

Agent	Class	Subtype	Trial phase	n	Median follow- up	ORR	CR	Median PFS	Median OS	Reference
Pralatrexate	Antifolate	PTCL	II	111	18 months	29%	11%	3.5 months	14.5 months	36
Romidepsin	HDAC inhibitor	PTCL	II	130	22.3 months	25%	15%	4 months	11.3 months	40
Romidepsin	HDAC inhibitor	PTCL	II	45	—	38%	18%	—	—	41
Belinostat	HDAC inhibitor	PTCL	II	129	—	25.8%	10.8%	1.6 months	7.9 months	42
Chidamide	HDAC inhibitor	PTCL	II	83	29 months	28%	14%	2.1 months	21.4 months	43
Brentuximab vedotin	Anti-CD30 antibody drug conjugate	ALCL	II	58	71.4 months	86%	57%	20 months	Not reached	44, 54
Crizotinib	Tyrosine kinase inhibitor	ALK- positive ALCL	II	9	—	90.9%	100%	—	—	47
Duvelisib	PI3K-δ and PI3K-γ inhibitor	PTCL	I	16	—	50%	19%	8.3 months	8.4 months	48
Mogamulizumab	Anti-CCR4 antibody	CCR4- positive PTCL	II	29	—	34%	17%	2.0 months	14.2 months	51
Nivolumab	Anti-PD-1 antibody	PTCL	I	5	44 weeks	40%	0	14 weeks	—	55

Abbreviations: ALCL, anaplastic large cell lymphoma; ALK, anaplastic lymphoma kinase; CCR4, CC chemokine receptor 4; CR, complete response; HDAC, histone deacetylase inhibitor; ORR, objective response rate; OS, overall survival; PD-1, programmed death-1; PFS, progression-free survival; PI3K, phosphatidylinositol 3-kinase; PTCL, peripheral T-cell lymphoma.

Pralatrexate, an antifolate, was examined in a phase II study in which 111 patients with PTCL who had progressed after at least one line of prior therapy were treated weekly for 6 weeks in a 7-week cycle. Response rate was 29%, which included 11% complete responses and 18% partial responses. Median PFS and OS were 3.5 and 14.5 months, respectively ^36^. While this study led to its accelerated approval, further studies have confirmed the promising effects of pralatrexate in RR-PTCL ^37– 39^. Romidepsin, an HDAC inhibitor, has also been studied as a single agent in this setting with ORR of 25–38%, with the median duration of response ranging from 8.9–28 months across studies ^40, 41^. Belinostat, a pan-HDAC inhibitor, was also examined in a phase II trial of 129 patients with a median of two prior systemic therapies. The ORR was 25.8% with a median duration of response of 13.6 months ^42^. While the response rate to these agents is modest, the duration of response can be sustained in many cases, reflecting a need for predictive markers to identify those patients most likely to benefit. Chidamide, an oral HDAC inhibitor, has also been studied in a phase II trial of RR-PTCL. A total of 83 patients were enrolled with an ORR of 28%, median PFS of 2.1 months, and median OS of 21.4 months ^43^. The anti-CD30 antibody drug conjugate brentuximab vedotin has also shown promising results as a single agent. Pro and colleagues reported their phase II study of 58 patients with RR-ALCL (the majority were ALK-negative), with a CR rate of 66% as per investigator assessment and 57% per central review. Those who had achieved a CR had an OS of 79% and PFS of 57% at 5 years ^44^, leading to approval by the FDA for this indication. Bartlett and colleagues explored the use of brentuximab vedotin retreatment in HL and systemic ALCL patients who had previously responded. While it was only a small group of eight systemic ALCL patients, 88% responded to retreatment. With brentuximab vedotin moving to the frontline for many patients, these results suggest that retreatment should be explored further ^45^.

Alisertib, a selective, small-molecule aurora A kinase (AAK) inhibitor, was studied in an open-label, phase III trial compared to investigator’s choice in patients with RR-PTCL. Options for the single-agent comparator included pralatrexate, gemcitabine, or romidepsin. While the ORR for alisertib was 33%, the study was stopped early owing to the low probability of alisertib achieving superior PFS with full enrollment, as the control arm ORR was 45%. There was some slight suggestion that alisertib may be better tolerated than its comparators, as 33% required dose modifications on the comparator arm versus 28% in the alisertib arm because of adverse effects ^46^. Interestingly, the ORR was 35% for gemcitabine, 43% for pralatrexate, and 61% for romidepsin, although the trial was not powered to study individual agents in the comparator arm.

There are also a number of other targeted agents at various stages of investigation in patients with PTCL. Crizotinib is an oral, small-molecule tyrosine kinase inhibitor with binding specificity for ALK and ROS1. This molecule is particularly interesting for ALK-positive ALCL, in which ALK overexpression is constitutively active because of the oncogenic translocation in these tumors. When administered to a small group of ALK-positive ALCL and ALK-positive diffuse large B-cell lymphoma RR patients, there was an ORR of 90.9% with 100% CR in the ALCL group. The 2-year PFS and OS were 63.7% and 72.7%, respectively ^47^. Duvelisib is another oral kinase inhibitor that inhibits PI3K-δ and PI3K-γ. It has been studied for safety and tolerability both as a single agent and in combination with other novel therapies in patients with PTCL ^48, 49^. Preliminary results of the dose-expansion phase of the PRIMO study, a phase II study of duvelisib monotherapy in RR-PTCL, suggest that duvelisib is clinically active in RR-PTCL with no unexpected toxicities. Further expansion phase data are still pending ^50^. Duvelisib has gained an orphan drug designation and fast track designation for PTCL patients who have received at least one prior therapy.

Mogamulizumab is a humanized anti-CCR4 antibody with potent antibody-dependent cellular toxicity. It has been studied in the phase II setting in CCR4-positive PTCL and cutaneous T-cell lymphomas. The overall response rate was 34%, with a median PFS of 2 months for the PTCL group ^51^. It has also been compared to investigator-chosen regimens in RR-ATLL with encouraging results in this disease with very poor outcomes ^52^. A European study has reported an ORR of only 11% with a disease control rate (CR, PR, and stable disease) of 46% ^53^. Given these encouraging results, further randomized studies are underway.

Immune checkpoint inhibitors have been studied in most lymphoma subtypes with potential direct anti-tumor effects as well as effects in the tumor microenvironment. While activity in some settings has been modest, anti-PD1 antibodies may be of particular appeal in lymphomas associated with EBV infection (including NK/T-cell lymphoma) as well as other settings, leading to upregulation of the PD1/PDL1 axis, as can be seen in Hodgkin’s lymphoma (HL). A phase I study examined the use of nivolumab in various hematologic malignancies, including five patients with PTCL in which two patients achieved a partial response ^55^. A phase II study of 18 patients with RR T-cell lymphoma treated with pembrolizumab was closed early following a preplanned futility analysis in which 18 enrolled patients had an ORR of 33% ^56^. Further study of immune checkpoint inhibitors in T-cell lymphoma is clearly warranted, as there have also been some findings of hyperprogression in various subtypes. It is hypothesized that this rapid disease progression may be related to PD-1 acting as a tumor suppressor in some tumor types, further highlighting the need for clinical studies in biologically distinct subgroups ^57, 58^.

Combination strategies also warrant evaluation, whether this involves novel agents in combination with chemotherapy or novel doublets. Chemotherapy combination approaches include the addition of brentuximab vedotin to bendamustine (given the activity of the antibody drug conjugate in ALCL and the safety of the combination from the phase I evaluation in HL), which has shown early signs of activity in CD30-positive PTCL. Studies of immune checkpoint inhibitors in combination with chemotherapy are also underway. Given synergy in pre-clinical models, pralatrexate was combined with romidepsin in a phase I study to determine safety, tolerability, and maximum tolerated doses in RR lymphomas. The drug combination was found to be safe and reasonably tolerated with an ORR in the RR-PTCL subgroup of 71% ^59^. Given these promising findings, a phase II study was initiated and is ongoing. Horwitz and colleagues described a small, parallel phase I study of RR T-cell lymphoma patients, in which there were 21 evaluable PTCL patients who received romidepsin and duvelisib (ORR 55% and CR 27%) or bortezomib with duvelisib (ORR 36% and CR 21%). Transaminitis limited the tolerability of bortezomib with duvelisib, but, given the tolerability and activity of romidepsin and duvelisib, an expansion of that cohort is planned ^60^. Other combinations of novel treatments that are in the early stages of evaluation include pralatrexate with bortezomib ^61^ and romidepsin with pembrolizumab ^62^.

## Cellular therapy

Chimeric antigen receptor (CAR)-T cell immunotherapies have shown strong results in the treatment of B-cell malignancies. Targeting T-cell lymphomas with CAR-T cell therapies has some concern given the concept of T-cell fratricide. Anti-CD30 CAR-T cell treatments are in development and have been trialed in small studies thus far. Wang and colleagues included one patient with cutaneous ALCL, and Ramos and colleagues included one patient with cutaneous ALCL and one with systemic ALCL in their series. Grover and colleagues describe their phase Ib/II anti-CD30 CAR-T trial of 24 patients (including one with EATL and one with Sezary syndrome). When using a bendamustine and fludarabine lymphodepleting regimen in this study, they showed significant anti-tumor activity. The majority of patients in these studies had HL. These trials are preliminary but show good tolerability and some modest effects ^63– 65^. Hill and colleagues presented the preliminary results of a phase I dose escalation study of a CD5-directed CAR-T cell therapy in RR T-cell leukemia and lymphoma patients as a bridge to allo-SCT ^66^. They treated nine patients (four patients with T-NHL) and showed that treatment is safe and does not appear to induce T-cell aplasia. Some clinical responses were seen in this small and heavily pretreated population. The National Cancer Institute has an upcoming phase I trial assessing the safety and feasibility of an anti-CD30 CAR-T cell treatment in advanced CD30-expressing lymphomas, including ALCL and PTCL-NOS. Other early phase anti-CD30 CAR-T cell studies are currently recruiting ^67^. Also upcoming is a phase I/II study assessing an anti-T cell receptor β-chain constant domain 1 (TRBC1) CAR-T cell therapy in RR, TRBC1-expressing PTCL-NOS, AITL, and ALCL. There are two genes associated with the β-gene constant region of the T cell receptor, *TRBC1* and *TRBC2.* A group of normal T cells will therefore have a mixture of cells, as some express *TRBC1* and others express *TRBC2*. Clonality among a group of malignant T cells will therefore result in exclusive expression of one constant domain, making it an attractive target ^68^. These results and others will certainly be helpful to start clarifying the role that CAR-T cell immunotherapy will have in treating patients with RR-PTCL.

## Evolving approaches to PTCL

### Translational insights and developing effective treatment strategies

PTCL remains a clinically heterogeneous group of diseases, and therapy can only improve from studies that provide biologic insights into these tumors and further identify distinct lymphoma subtypes and targetable alterations. The identification of chromosomal abnormalities has been important in defining specific disease entities. Constitutively active ALK overexpression associated with a genetic translocation in some ALCLs has long been associated with a better prognosis ^69^. Now a distinct entity, ALK-negative ALCLs appear to have further cytogenetic subtypes that manifest varying clinical behavior, although further study is needed ^70– 72^. Newer biologic insights have identified a common T-follicular helper (TFH) cell signature among AITL, follicular T-cell lymphoma (FTCL), and in some cases of PTCL-NOS, leading to a new umbrella category of Nodal T-cell Lymphomas with TFH phenotype in the 2016 WHO classification ^73^. A number of the common genetic changes including *TET2*, *IDH2*, *DNMT3A, RHOA*, and others are also frequently altered in myeloid neoplasms, most commonly acute myeloid leukemias (AML) and myelodysplastic syndromes (MDS) ^73– 76^. Azacitidine, a hypomethylating agent, as well as enasidenib (IDH2 inhibitor) and ivosidenib (IDH1 inhibitor) via their effects on these altered pathways led to improved outcomes in MDS ^77^ and AML ^78, 79^. Case reports of patients with concomitant AITL and a myeloid neoplasm, as well as a retrospective series of 12 AITL patients, seven of whom did not have concomitant myeloid disease, were treated with azacitidine. With an ORR of 75% in the series and four of the seven patients without a concomitant myeloid neoplasm responding, these data suggest there may be a lymphoma response that is not restricted to those patients with an associated myeloid disease ^80– 83^. This has led to further study, including a phase III trial examining the use of oral azacitidine compared to investigator’s choice in RR-AITL which is currently accruing. A phase I study of oral azacitidine with romidepsin in advanced lymphoid malignancies had an ORR of 73% ^84^. A trial of enasidenib in advanced solid tumors including IDH2-mutated AITL has been completed but is yet to be reported. While gene expression profiling has led to the identification of some similarities among subtypes, it has also confirmed the biologic heterogeneity among others. Overexpression of *GATA3*, *TBX21*, and cytotoxic genes characterized a cohort of PTCL cases into distinct groups with varying clinical behavior ^85^, and next-generation sequencing studies have further characterized the genetic alterations in epigenetic modifiers, signaling genes, and tumor suppressors.

Targeted approaches which have shown promise in RR-PTCL have subsequently been brought for evaluation earlier in the disease course, and to date there have been only a handful of such agents. The most successful example is the development of brentuximab vedotin in CD30-positive T-cell lymphomas. Given results of the drug’s efficacy as a single agent in the RR setting and tolerability in combination with cyclophosphamide, doxorubicin, and prednisone (A+CHP) in a phase I study, the ECHELON-2 study was performed, leading to the registration of brentuximab vedotin as part of primary therapy in CD30-positive PTCL. Overall survival and PFS improvement was demonstrated in the brentuximab combination arm (HR 0.66, *P* = 0.0244 and HR 0.71, *P* = 0.0110, respectively) with an acceptable toxicity profile ^5^. Further studies are needed with adequate power to explore its use in all CD30-positive subtypes given the majority of ALCL patients in this trial. A similar trial comparing CHOP with romidepsin in combination with CHOP has been accrued and results are awaited.

Rational drug development supports the development of these highly targeted agents in patient subsets that are known to express the drug target, although it is certainly possible that these agents could have additional mechanisms of action that could lead to activity through off-target effects, potentially directly on the tumor or on the microenvironment. Randomized approaches are also required to evaluate active regimens in the RR setting. Multiple questions remain, including the combination of chemotherapy with novel agents, novel doublets, and integrating targeted agents into SCT strategies. Relying on historic approaches that have not been well defined by randomized data appears less appealing when considering the unfavorable outcomes for these patients despite dose-intensive strategies. Given the variety of agents and variety of lymphoma subsets that may be relevant to each drug, clinical trial designs incorporating multiple histologies or tumors with a focus on the drug target, such as basket or umbrella studies, are clearly needed to feasibly conduct these trials in uncommon disease indications. These more efficient trial designs would allow trials to open and maximize accrual and minimize costs in individual centers. This would make opening trials in RR-PTCL more appealing for smaller lymphoma programs and facilitate prompt accrual for investigators and sponsors alike.

## Summary and conclusions

PTCL is a heterogeneous group of rare NHLs for which outcomes remain rather poor. Standard frontline therapy currently remains traditional chemotherapy-based regimens with or without ASCT in first remission for those who are candidates. Only recently has the CD30-targeted agent brentuximab vedotin started to challenge this paradigm. The WHO 2016 update has improved the classification of T-cell lymphomas into relevant biological and clinical subtypes, which will hopefully allow further elucidation into the specific biology that drives oncogenesis, tumor suppression, proliferation, and drug responses. This will lead to the identification of further biomarkers, relevant drug targets, and more accurate biological and clinical classifications, which will allow improved assessment of new protocols.

Significant advances have been made with a number of promising novel and targeted agents in the RR setting. However, studies remain challenging when grouping these rare, heterogeneous diseases together, which results in the possibility of missed successes (or failures) among certain disease subtypes. Selecting the patient or disease subgroups most likely to benefit and eventually moving these drugs earlier in the disease course will require elegant trial design using novel biomarkers and improved disease classification.

Further exploration into the biological heterogeneity of disease subtypes will allow the development of rationally designed therapeutic agents, with directed clinical trials to specific subtypes or patients who are most likely to respond. Unique trial designs with multiple arms and baskets will allow the study of these targeted agents in rare disease subtypes. The rational combination of novel agents and/or chemotherapy will be possible as more knowledge is gleaned regarding the biological mechanisms of action and which patients are most likely to benefit. It is with these improved techniques and personalized approaches that we can aim to improve outcomes for patients with aggressive T-cell lymphomas.

## References

[1] Vose J Armitage J Weisenburger D: International peripheral T-cell and natural killer/T-cell lymphoma study: Pathology findings and clinical outcomes. *J Clin Oncol.* 2008;26(25):4124–30. 10.1200/JCO.2008.16.4558 18626005

[2] Schmitz N Trümper L Ziepert M: Treatment and prognosis of mature T-cell and NK-cell lymphoma: An analysis of patients with T-cell lymphoma treated in studies of the German High-Grade Non-Hodgkin Lymphoma Study Group. *Blood.* 2010;116(18):3418–25. 10.1182/blood-2010-02-270785 20660290

[3] Sieniawski M Angamuthu N Boyd K: Evaluation of enteropathy-associated T-cell lymphoma comparing standard therapies with a novel regimen including autologous stem cell transplantation. *Blood.* 2010;115(18):3664–70. 10.1182/blood-2009-07-231324 20197551

[4] Phillips EH Lannon MM Lopes A: High-dose chemotherapy and autologous stem cell transplantation in enteropathy-associated and other aggressive T-cell lymphomas: A UK NCRI/Cancer Research UK Phase II Study. *Bone Marrow Transplant.* 2019;54(3):465–8. 10.1038/s41409-018-0294-2 30104718

[5] Horwitz S O'Connor OA Pro B: Brentuximab vedotin with chemotherapy for CD30-positive peripheral T-cell lymphoma (ECHELON-2): A global, double-blind, randomised, phase 3 trial. *Lancet.* 2019;393(10168):229–40. 10.1016/S0140-6736(18)32984-2 30522922 PMC6436818

[6] Savage KJ Horwitz SM Advani RH: An Exploratory Analysis of Brentuximab Vedotin Plus CHP (A+CHP) in the Frontline Treatment of Patients with CD30+ Peripheral T-Cell Lymphomas (ECHELON-2): Impact of Consolidative Stem Cell Transplant. *Blood.* 2019;134(Supplement_1):464 10.1182/blood-2019-122781

[7] Biasoli I Cesaretti M Bellei M: Dismal outcome of T-cell lymphoma patients failing first-line treatment: Results of a population-based study from the Modena Cancer Registry. *Hematol Oncol.* 2015;33(3):147–51. 10.1002/hon.2144 24777784

[8] Mak V Hamm J Chhanabhai M: Survival of Patients With Peripheral T-Cell Lymphoma After First Relapse or Progression: Spectrum of Disease and Rare Long-Term Survivors. *J Clin Oncol.* 2013;31(16):1970–6. 10.1200/JCO.2012.44.7524 23610113

[9] Chihara D Fanale MA Miranda RN: The survival outcome of patients with relapsed/refractory peripheral T-cell lymphoma-not otherwise specified and angioimmunoblastic T-cell lymphoma. *Br J Haematol.* 2017;176(5):750–8. 10.1111/bjh.14477 27983760 PMC5836501

[10] Bellei M Foss FM Shustov AR: The outcome of peripheral T-cell lymphoma patients failing first-line therapy: A report from the prospective, International T-Cell Project. *Haematologica.* 2018;103(7):1191–7. 10.3324/haematol.2017.186577 29599200 PMC6029527

[11] Barrington SF Mikhaeel NG Kostakoglu L: Role of imaging in the staging and response assessment of lymphoma: Consensus of the International Conference on Malignant Lymphomas Imaging Working Group. *J Clin Oncol.* 2014;32(27):3048–58. 10.1200/JCO.2013.53.5229 25113771 PMC5015423

[12] Cheson BD Fisher RI Barrington SF: Recommendations for Initial Evaluation, Staging, and Response Assessment of Hodgkin and Non-Hodgkin Lymphoma: The Lugano Classification. *J Clin Oncol.* 2014;32(27):3059–67. 10.1200/JCO.2013.54.8800 25113753 PMC4979083

[13] Cottereau AS El-Galaly TC Becker S: Predictive Value of PET Response Combined with Baseline Metabolic Tumor Volume in Peripheral T-Cell Lymphoma Patients. *J Nucl Med.* 2018;59(4):589–95. 10.2967/jnumed.117.193946 28864629

[14] El-Galaly TC Pedersen MB Hutchings M: Utility of interim and end-of-treatment PET/CT in peripheral T-cell lymphomas: A review of 124 patients. *Am J Hematol.* 2015;90(11):975–80. 10.1002/ajh.24128 26201505

[15] Gurion R Bernstine H Domachevsky L: Utility of PET-CT for Evaluation of Patients With Peripheral T-cell Lymphoma. *Clin Lymphoma Myeloma Leuk.* 2018;18(10):687–91. 10.1016/j.clml.2018.06.022 30017596

[16] Jung SH Ahn JS Kim YK: Prognostic significance of interim PET/CT based on visual, SUV-based, and MTV-based assessment in the treatment of peripheral T-cell lymphoma. *BMC Cancer.* 2015;15:198. 10.1186/s12885-015-1193-1 25879747 PMC4379548

[17] Shea L Liu J Cashen A: Prognostic significance of 18Ffluorodeoxyglucose-positron emission tomography in peripheral T-cell lymphoma treated with stem cell transplantation: A retrospective analysis. *Leuk Lymphoma.* 2015;56(1):256–9. 10.3109/10428194.2014.914194 24730536 PMC4890163

[18] Gallamini A Stelitano C Calvi R: Peripheral T-cell lymphoma unspecified (PTCL-U): A new prognostic model from a retrospective multicentric clinical study. *Blood.* 2004;103(7):2474–9. 10.1182/blood-2003-09-3080 14645001

[19] Skamene T Crump M Savage KJ: Salvage chemotherapy and autologous stem cell transplantation for peripheral T-cell lymphoma: A subset analysis of the Canadian Cancer Trials Group LY.12 randomized phase 3 study. *Leuk Lymphoma.* 2017;58(10):2319–27. 10.1080/10428194.2017.1312379 28504033

[20] Park BB Kim WS Suh C: Salvage chemotherapy of gemcitabine, dexamethasone, and cisplatin (GDP) for patients with relapsed or refractory peripheral T-cell lymphomas: A consortium for improving survival of lymphoma (CISL) trial. *Ann Hematol.* 2015;94(11):1845–51. 10.1007/s00277-015-2468-y 26251158

[21] Kogure Y Yoshimi A Ueda K: Modified ESHAP regimen for relapsed/refractory T cell lymphoma: A retrospective analysis. *Ann Hematol.* 2015;94(6):989–94. 10.1007/s00277-015-2309-z 25687839

[22] Zelenetz AD Hamlin P Kewalramani T: Ifosfamide, carboplatin, etoposide (ICE)-based second-line chemotherapy for the management of relapsed and refractory aggressive non-Hodgkin's lymphoma. *Ann Oncol.* 2003;14 Suppl 1:i5–10. 10.1093/annonc/mdg702 12736224

[23] Yang YT Tai CJ Chen C: Highly Diverse Efficacy of Salvage Treatment Regimens for Relapsed or Refractory Peripheral T-Cell Lymphoma: A Systematic Review. *PLoS One.* 2016;11(10):e0161811. 10.1371/journal.pone.0161811 27711130 PMC5053427

[24] Stuver RN Khan N Schwartz M: Single agents vs combination chemotherapy in relapsed and refractory peripheral T-cell lymphoma: Results from the comprehensive oncology measures for peripheral T-cell lymphoma treatment (COMPLETE) registry. *Am J Hematol.* 2019;94(6):641–9. 10.1002/ajh.25463 30896890 PMC7928240

[25] Kharfan-Dabaja MA Kumar A Ayala E: Clinical Practice Recommendations on Indication and Timing of Hematopoietic Cell Transplantation in Mature T Cell and NK/T Cell Lymphomas: An International Collaborative Effort on Behalf of the Guidelines Committee of the American Society for Blood and Marrow Transplantation. *Biol Blood Marrow Transplant.* 2017;23(11):1826–38. 10.1016/j.bbmt.2017.07.027 28797780

[26] Feyler S Prince HM Pearce R: The role of high-dose therapy and stem cell rescue in the management of T-cell malignant lymphomas: A BSBMT and ABMTRR study. *Bone Marrow Transplant.* 2007;40(5):443–50. 10.1038/sj.bmt.1705752 17589529

[27] Jantunen E Wiklund T Juvonen E: Autologous stem cell transplantation in adult patients with peripheral T-cell lymphoma: A nation-wide survey. *Bone Marrow Transplant.* 2004;33(4):405–10. 10.1038/sj.bmt.1704367 14676776

[28] Puig N Wang L Seshadri T: Treatment response and overall outcome of patients with relapsed and refractory peripheral T-cell lymphoma compared to diffuse large B-cell lymphoma. *Leuk Lymphoma.* 2013;54(3):507–13. 10.3109/10428194.2012.719615 22897731

[29] Rodríguez J Conde E Gutiérrez A: The adjusted International Prognostic Index and beta-2-microglobulin predict the outcome after autologous stem cell transplantation in relapsing/refractory peripheral T-cell lymphoma. *Haematologica.* 2007;92(8):1067–74. 10.3324/haematol.11173 17640855

[30] Song KW Mollee P Keating A: Autologous stem cell transplant for relapsed and refractory peripheral T-cell lymphoma: Variable outcome according to pathological subtype. *Br J Haematol.* 2003;120(6):978–85. 10.1046/j.1365-2141.2003.04203.x 12648067

[31] Smith SM Burns LJ van Besien K: Hematopoietic cell transplantation for systemic mature T-cell non-Hodgkin lymphoma. *J Clin Oncol.* 2013;31(25):3100–9. 10.1200/JCO.2012.46.0188 23897963 PMC3753702

[32] Chen AI McMillan A Negrin RS: Long-Term Results Of Autologous Hematopoietic Cell Transplantation For Peripheral T Cell Lymphoma: The Stanford Experience. *Biol Blood Marrow Transplant.* 2008;14(7):741–7. 10.1016/j.bbmt.2008.04.004 18541192 PMC2980839

[33] Philip T Guglielmi C Hagenbeek A: Autologous bone marrow transplantation as compared with salvage chemotherapy in relapses of chemotherapy-sensitive non-Hodgkin's lymphoma. *N Engl J Med.* 1995;333(23):1540–5. 10.1056/NEJM199512073332305 7477169

[34] El-Asmar J Reljic T Ayala E: Efficacy of High-Dose Therapy and Autologous Hematopoietic Cell Transplantation in Peripheral T Cell Lymphomas as Front-Line Consolidation or in the Relapsed/Refractory Setting: A Systematic Review/Meta-Analysis. *Biol Blood Marrow Transplant.* 2016;22(5):802–14. 10.1016/j.bbmt.2015.12.004 26713431

[35] Schmitz N Lenz G Stelljes M: Allogeneic hematopoietic stem cell transplantation for T-cell lymphomas. *Blood.* 2018;132(3):245–53. 10.1182/blood-2018-01-791335 29699989

[36] O'Connor OA Pro B Pinter-Brown L: Pralatrexate in Patients With Relapsed or Refractory Peripheral T-Cell Lymphoma: Results From the Pivotal PROPEL Study. *J Clin Oncol.* 2011;29(9):1182–9. 10.1200/JCO.2010.29.9024 21245435 PMC3083873

[37] Maruyama D Nagai H Maeda Y: Phase I/II study of pralatrexate in Japanese patients with relapsed or refractory peripheral T-cell lymphoma. *Cancer Sci.* 2017;108(10):2061–8. 10.1111/cas.13340 28771889 PMC5623731

[38] Hong X Song Y Huang H: Pralatrexate in Chinese Patients with Relapsed or Refractory Peripheral T-cell Lymphoma: A Single-arm, Multicenter Study. *Target Oncol.* 2019;14(2):149–58. 10.1007/s11523-019-00630-y 30904980 PMC6453867

[39] O'Connor OA Marchi E Volinn W: Strategy for Assessing New Drug Value in Orphan Diseases: An International Case Match Control Analysis of the PROPEL Study. *JNCI Cancer Spectr.* 2018;2(4):pky038. 10.1093/jncics/pky038 31360868 PMC6649793

[40] Coiffier B Pro B Prince HM: Romidepsin for the treatment of relapsed/refractory peripheral T-cell lymphoma: Pivotal study update demonstrates durable responses. *J Hematol Oncol.* 2014;7:11. 10.1186/1756-8722-7-11 24456586 PMC4016573

[41] Piekarz RL Frye R Prince HM: Phase 2 trial of romidepsin in patients with peripheral T-cell lymphoma. *Blood.* 2011;117(22):5827–34. 10.1182/blood-2010-10-312603 21355097 PMC3112033

[42] O'Connor OA Horwitz S Masszi T: Belinostat in Patients With Relapsed or Refractory Peripheral T-Cell Lymphoma: Results of the Pivotal Phase II BELIEF (CLN-19) Study. *J Clin Oncol.* 2015;33(23):2492–9. 10.1200/JCO.2014.59.2782 26101246 PMC5087312

[43] Shi Y Dong M Hong X: Results from a multicenter, open-label, pivotal phase II study of chidamide in relapsed or refractory peripheral T-cell lymphoma. *Ann Oncol.* 2015;26(8):1766–71. 10.1093/annonc/mdv237 26105599

[44] Pro B Advani R Brice P: Five-year results of brentuximab vedotin in patients with relapsed or refractory systemic anaplastic large cell lymphoma. *Blood.* 2017;130(25):2709–17. 10.1182/blood-2017-05-780049 28974506 PMC5746164

[45] Bartlett NL Chen R Fanale MA: Retreatment with brentuximab vedotin in patients with CD30-positive hematologic malignancies. *J Hematol Oncol.* 2014;7:24. 10.1186/1756-8722-7-24 24642247 PMC3994656

[46] O'Connor OA Özcan M Jacobsen ED: Randomized Phase III Study of Alisertib or Investigator's Choice (Selected Single Agent) in Patients With Relapsed or Refractory Peripheral T-Cell Lymphoma. *J Clin Oncol.* 2019;37(8):613–23. 10.1200/JCO.18.00899 30707661 PMC6494247

[47] Passerini CG Farina F Stasia A: Crizotinib in Advanced, Chemoresistant Anaplastic Lymphoma Kinase–Positive Lymphoma Patients. *J Natl Cancer Inst.* 2014;106(2):djt378. 10.1093/jnci/djt378 24491302

[48] Horwitz SM Koch R Porcu P: Activity of the PI3K-δ,γ inhibitor duvelisib in a phase 1 trial and preclinical models of T-cell lymphoma. *Blood.* 2018;131(8):888–98. 10.1182/blood-2017-08-802470 29233821 PMC5824337

[49] Flinn IW O’Brien S Kahl B: Duvelisib, a novel oral dual inhibitor of PI3K-δ,γ, is clinically active in advanced hematologic malignancies. *Blood.* 2018;131(8):877–87. 10.1182/blood-2017-05-786566 29191916 PMC6033052

[50] Horwitz SM Mehta-Shah N Pro B: Dose Optimization of Duvelisib in Patients with Relapsed or Refractory Peripheral T-Cell Lymphoma from the Phase 2 Primo Trial: Selection of Regimen for the Dose-Expansion Phase. *Blood.* 2019;134(Supplement_1):1567 10.1182/blood-2019-121401

[51] Ogura M Ishida T Hatake K: Multicenter phase II study of mogamulizumab (KW-0761), a defucosylated anti-cc chemokine receptor 4 antibody, in patients with relapsed peripheral T-cell lymphoma and cutaneous T-cell lymphoma. *J Clin Oncol.* 2014;32(11):1157–63. 10.1200/JCO.2013.52.0924 24616310

[52] Phillips AA Fields PA Hermine O: Mogamulizumab *versus* investigator's choice of chemotherapy regimen in relapsed/refractory adult T-cell leukemia/lymphoma. *Haematologica.* 2019;104(5):993–1003. 10.3324/haematol.2018.205096 30573506 PMC6518882

[53] Zinzani PL Karlin L Radford J: European phase II study of mogamulizumab, an anti-CCR4 monoclonal antibody, in relapsed/refractory peripheral T-cell lymphoma. *Haematologica.* 2016;101(10):e407–e410. 10.3324/haematol.2016.146977 27418646 PMC5046662

[54] Pro B Advani R Brice P: Brentuximab Vedotin (SGN-35) in Patients With Relapsed or Refractory Systemic Anaplastic Large-Cell Lymphoma: Results of a Phase II Study. *J Clin Oncol.* 2012;30(18):2190–6. 10.1200/JCO.2011.38.0402 22614995

[55] Lesokhin AM Ansell SM Armand P: Nivolumab in Patients With Relapsed or Refractory Hematologic Malignancy: Preliminary Results of a Phase Ib Study. *J Clin Oncol.* 2016;34(23):2698–704. 10.1200/JCO.2015.65.9789 27269947 PMC5019749

[56] Barta SK Zain J MacFarlane AW: Phase II Study of the PD-1 Inhibitor Pembrolizumab for the Treatment of Relapsed or Refractory Mature T-cell Lymphoma. *Clin Lymphoma Myeloma Leuk.* 2019;19(6):356–364.e3. 10.1016/j.clml.2019.03.022 31029646 PMC7433797

[57] Wartewig T Kurgyis Z Keppler S: PD-1 is a haploinsufficient suppressor of T cell lymphomagenesis. *Nature.* 2017;552(7683):121–5. 10.1038/nature24649 29143824 PMC5821214

[58] Ratner L Waldmann TA Janakiram M: Rapid Progression of Adult T-Cell Leukemia-Lymphoma after PD-1 Inhibitor Therapy. *N Engl J Med.* 2018;378(20):1947–8. 10.1056/NEJMc1803181 29768155

[59] Amengual JE Lichtenstein R Lue J: A phase 1 study of romidepsin and pralatrexate reveals marked activity in relapsed and refractory T-cell lymphoma. *Blood.* 2018;131(4):397–407. 10.1182/blood-2017-09-806737 29141948 PMC5790128

[60] Horwitz SM Moskowitz AJ Jacobsen ED: The Combination of Duvelisib, a PI3K-δ,γ Inhibitor, and Romidepsin Is Highly Active in Relapsed/Refractory Peripheral T-Cell Lymphoma with Low Rates of Transaminitis: Results of Parallel Multicenter, Phase 1 Combination Studies with Expansion Cohorts. *Blood.* 2018;132(Supplement 1):683 10.1182/blood-2018-99-115241

[61] Lee SS Jung SH Ahn JS: Pralatrexate in Combination with Bortezomib for Relapsed or Refractory Peripheral T Cell Lymphoma in 5 Elderly Patients. *J Korean Med Sci.* 2016;31(7):1160–3. 10.3346/jkms.2016.31.7.1160 27366017 PMC4901011

[62] Iyer SP Neelapu SS Burns E: A Phase I/II Study to Examine the Safety and Efficacy of Pembrolizumab 200 Mg Fixed Dose Administered Every 3 Weeks (Q3W) in Combination with Romidepsin in Relapsed or Refractory Peripheral T-Cell Lymphoma (PTCL). *Blood.* 2019;134(Supplement_1):1546 10.1182/blood-2019-132278

[63] Wang CM Wu ZQ Wang Y: Autologous T Cells Expressing CD30 Chimeric Antigen Receptors for Relapsed or Refractory Hodgkin Lymphoma: An Open-Label Phase I Trial. *Clin Cancer Res.* 2017;23(5):1156–66. 10.1158/1078-0432.CCR-16-1365 27582488

[64] Ramos CA Ballard B Zhang H: Clinical and immunological responses after CD30-specific chimeric antigen receptor–redirected lymphocytes. *J Clin Invest.* 2017;127(9):3462–71. 10.1172/JCI94306 28805662 PMC5669573

[65] Grover NS Park SI Ivanova A: A Phase Ib/II Study of Anti-CD30 Chimeric Antigen Receptor T Cells for Relapsed/Refractory CD30+ Lymphomas. *Biol Blood Marrow Transplant.* 2019;25(3):S66 10.1016/j.bbmt.2018.12.149

[66] Hill LC Rouce RH Smith TS: Safety and Anti-Tumor Activity of CD5 CAR T-Cells in Patients with Relapsed/Refractory T-Cell Malignancies. *Blood.* 2019;134(Supplement_1):199 10.1182/blood-2019-129559 31064751

[67] Grover NS Savoldo B: Challenges of driving CD30-directed CAR-T cells to the clinic. *BMC Cancer.* 2019;19(1):203. 10.1186/s12885-019-5415-9 30841880 PMC6404322

[68] Maciocia PM Wawrzyniecka PA Philip B: Targeting the T cell receptor β-chain constant region for immunotherapy of T cell malignancies. *Nat Med.* 2017;23(12):1416–23. 10.1038/nm.4444 29131157

[69] Savage KJ Harris NL Vose JM: ALK- anaplastic large-cell lymphoma is clinically and immunophenotypically different from both ALK ^+^ ALCL and peripheral T-cell lymphoma, not otherwise specified: Report from the International Peripheral T-Cell Lymphoma Project. *Blood.* 2008;111(12):5496–504. 10.1182/blood-2008-01-134270 18385450

[70] Parrilla Castellar ER Jaffe ES: ALK-negative anaplastic large cell lymphoma is a genetically heterogeneous disease with widely disparate clinical outcomes. *Blood.* 2014;124(9):1473–80. 10.1182/blood-2014-04-571091 24894770 PMC4148769

[71] Hapgood G Ben-Neriah S Mottok A: Identification of high-risk *DUSP22*-rearranged ALK-negative anaplastic large cell lymphoma. *Br J Haematol.* 2019;186(3):e28–e31. 10.1111/bjh.15860 30873584 PMC7679007

[72] King RL Dao LN McPhail ED: Morphologic Features of ALK-negative Anaplastic Large Cell Lymphomas With *DUSP22* Rearrangements. *Am J Surg Pathol.* 2016;40(1):36–43. 10.1097/PAS.0000000000000500 26379151 PMC4834837

[73] Swerdlow SH Campo E Pileri SA: The 2016 revision of the World Health Organization classification of lymphoid neoplasms. *Blood.* 2016;127(20):2375–90. 10.1182/blood-2016-01-643569 26980727 PMC4874220

[74] Lemonnier F Couronné L Parrens M: Recurrent *TET2* mutations in peripheral T-cell lymphomas correlate with T _FH_-like features and adverse clinical parameters. *Blood.* 2012;120(7):1466–9. 10.1182/blood-2012-02-408542 22760778

[75] Sakata-Yanagimoto M Enami T Yoshida K: Somatic *RHOA* mutation in angioimmunoblastic T cell lymphoma. *Nat Genet.* 2014;46(2):171–5. 10.1038/ng.2872 24413737

[76] Cairns RA Iqbal J Lemonnier F: *IDH2* mutations are frequent in angioimmunoblastic T-cell lymphoma. *Blood.* 2012;119(8):1901–3. 10.1182/blood-2011-11-391748 22215888 PMC3293643

[77] Fenaux P Mufti GJ Hellstrom-Lindberg E: Efficacy of azacitidine compared with that of conventional care regimens in the treatment of higher-risk myelodysplastic syndromes: A randomised, open-label, phase III study. *Lancet Oncol.* 2009;10(3):223–32. 10.1016/S1470-2045(09)70003-8 19230772 PMC4086808

[78] DiNardo CD Stein EM de Botton S: Durable Remissions with Ivosidenib in IDH1-Mutated Relapsed or Refractory AML. *N Engl J Med.* 2018;378(25):2386–98. 10.1056/NEJMoa1716984 29860938

[79] Stein EM DiNardo CD Pollyea DA: Enasidenib in mutant *IDH2* relapsed or refractory acute myeloid leukemia. *Blood.* 2017;130(6):722–31. 10.1182/blood-2017-04-779405 28588020 PMC5572791

[80] Cheminant M Bruneau J Kosmider O: Efficacy of 5-azacytidine in a *TET2* mutated angioimmunoblastic T cell lymphoma. *Br J Haematol.* 2015;168(6):913–6. 10.1111/bjh.13170 25312805

[81] Lemonnier F Dupuis J Sujobert P: Treatment with 5-azacytidine induces a sustained response in patients with angioimmunoblastic T-cell lymphoma. *Blood.* 2018;132(21):2305–9. 10.1182/blood-2018-04-840538 30279227

[82] Saillard C Guermouche H Derrieux C: Response to 5-azacytidine in a patient with *TET2*-mutated angioimmunoblastic T-cell lymphoma and chronic myelomonocytic leukaemia preceded by an EBV-positive large B-cell lymphoma. *Hematol Oncol.* 2017;35(4):864–8. 10.1002/hon.2319 27353473

[83] Tobiasson M Pandzic T Cavelier L: Angioimmunoblastic T-cell lymphoma and myelodysplastic syndrome with mutations in *TET2*, *DNMT3* and *CUX1* - azacitidine induces only lymphoma remission. *Leuk Lymphoma.* 2019;60(13):3316–9. 10.1080/10428194.2019.1627541 31204875

[84] O’Connor OA Falchi L Lue JK: Oral 5-azacytidine and romidepsin exhibit marked activity in patients with PTCL: A multicenter phase 1 study. *Blood.* 2019;134(17):1395–405. 10.1182/blood.2019001285 31471376

[85] Iqbal J Wright G Wang C: Gene expression signatures delineate biological and prognostic subgroups in peripheral T-cell lymphoma. *Blood.* 2014;123(19):2915–23. 10.1182/blood-2013-11-536359 24632715 PMC4014836

